# Hesitancy towards the Third Dose of COVID-19 Vaccine among the Younger Generation in Japan

**DOI:** 10.3390/ijerph19127041

**Published:** 2022-06-08

**Authors:** Mostafa Saidur Rahim Khan, Trinh Xuan Thi Nguyen, Sumeet Lal, Somtip Watanapongvanich, Yoshihiko Kadoya

**Affiliations:** School of Economics, Hiroshima University, 1-2-1 Kagamiyama, Higashihiroshima 7398525, Japan; xuantrinh93@gmail.com (T.X.T.N.); sumeetshivamlal@yahoo.com (S.L.); somtip.w@gmail.com (S.W.); ykadoya@hiroshima-u.ac.jp (Y.K.)

**Keywords:** COVID-19, booster dose, vaccine apprehension, Japan

## Abstract

The younger generation’s hesitancy towards the COVID-19 vaccine in Japan received significant attention during the early stages of vaccination. However, there is a lack of a comprehensive study in Japan that analyzes the apprehension towards the third dose of vaccine, commonly known as the booster dose, and its underlying causes. Using data from an online panel survey conducted by the Hiroshima Institute of Health Economics Research at Hiroshima University, we examined the severity of booster dose aversion among youths of different ages. Our findings indicate that a sizeable proportion of the Japanese population, particularly younger men, are hesitant to receive the booster dose. Furthermore, an inter-age group difference in booster dose aversion exists only among men. According to the probit regression results, subjective health status and future anxiety are associated with the booster vaccine hesitancy of men and women of various age groups. Moreover, few socioeconomic and behavioral factors like marital status, having children, household income and assets, and having a myopic view of the future, are also associated with the booster dose aversion among youths of certain ages. Given the diverse attitude of the younger generation, our findings suggest that public health authorities should develop effective communication strategies to reduce vaccine apprehension in the society.

## 1. Introduction

Hesitancy towards the COVID-19 vaccine has been a global concern since the approval of vaccines, which delays the achievement of herd immunity [1]. Despite the high efficacy of vaccines against the virus, several studies have documented the hesitancy towards the vaccination program in the early stages due to lack of trust in vaccines or governments, lack of information, conflict among authorities, issues of vaccine safety and effectiveness, and socioeconomic issues such as gender, age, education, ethnicity, attitude, political viewpoints, and so on [2,3,4,5,6,7,8,9]. The pursuit of a long-lasting immune response to the SARS-CoV-2 virus through a third dose of vaccine, commonly known as the booster dose, has been progressing globally. Major global health authorities advocated for the third dose of vaccine to be administered in the last quarter of 2021, first to immunocompromised people and then to the general public to boost immune response [10,11,12]. The need for a third dose stems from global evidence of waning immunity from the second dose of vaccine over time, as well as increased protection against newly emerged SARS-CoV-2 virus variants such as Delta and Omicron [13,14,15,16,17]. Importantly, evidence of an effective immune response and a lower risk of disease severity has been well established after the third dose of vaccine [18,19]. After the completion of the vaccination among priority groups, the mass vaccination program is now underway with the goal of including all eligible populations. However, similar to the initial phases of the vaccination program, hesitancy towards the third dose of vaccine still remains a concern for the health authority [20,21,22,23,24,25].

The evidence on the factors responsible for the hesitancy towards the third dose of vaccine is globally limited [20,21,24,25,26,27,28]. Moreover, the existing literature shows that the magnitude of hesitancy towards the third dose of the COVID-19 vaccine and its causes differ across the countries [21,24,25,26,27,28]. Lounis et al. [29] shows that vaccine acceptance rates range from 61.8% to 98% in the developed countries such as the USA, the UK, Japan, Germany, and Poland. Common factors that are responsible for the hesitancy towards the third dose of vaccine include younger age, being unmarried or single, lower education, and lower confidence in vaccines [20,23,25,26,27,28,29,30]. Moreover, several studies show that the uncertainty about the need for an additional dose, perceived effectiveness of vaccines, safety issues, and adverse side effects of initial doses are also responsible for booster vaccine hesitancy [4,22,31,32]. The inefficient communication strategy of the government is partly responsible for vaccine hesitancy as well [21,32]. However, Dubé and MacDonald [33] show that the hesitancy towards the COVID-19 vaccine varies broadly between countries and between groups with different socioeconomic and demographic characteristics.

Hesitancy towards the third dose of the COVID-19 vaccine is also an important issue for Japan due to previous evidence of vaccine hesitancy particularly among the younger generation [7,34,35,36,37]. However, a comprehensive nationwide study on the magnitude of hesitancy towards the third dose of vaccine in Japan and its associated factors is still lacking. Yoshida et al. [31] made an effort to study booster vaccine hesitancy in the Fukushima area and found that younger respondents with a higher antibody level were more likely to be vaccine hesitant. On the background of global evidence of varying magnitude of booster vaccine hesitancy and a lack of a comprehensive study in Japan, we investigate the hesitancy towards the third dose of the COVID-19 vaccine among younger Japanese populations across various age and gender strata. Moreover, we estimate the relationship between socio-economic factors and hesitancy towards the third dose of vaccine. Since most of the older populations (aged 65 and above) have already been vaccinated in Japan [38], we focus on younger populations (aged under 65). Moreover, despite the fact that the younger generation is the most vaccine-hesitant group [7,34,35,36,37], a detailed investigation on the reason behind their booster vaccine hesitancy is still missing. Our study contributes to the existing literature by elucidating the attitudes of younger populations towards the third dose of vaccine, which ultimately assists health authorities in developing an appropriate strategy to enroll all eligible populations in the vaccination program to achieve herd immunity in the society.

The remainder of this paper is organized as follows: Section 2 presents the data and methodology. The empirical results and discussion are presented in Section 3 and Section 4, respectively. Section 5 presents our conclusions.

## 2. Materials and Methods

### 2.1. Data

This study uses panel data from the Household Behavioral and Financial Survey, which was funded by Hiroshima University’s Hiroshima Institute of Health Economics Research (HiHER). Nikkei Research, a Japanese research firm, conducted the online survey. Their database is one of the largest in the country and represents the Japanese population from all socioeconomic backgrounds. The survey, which was conducted annually starting from February 2020, was aimed at the Japanese population aged 20 and above. The data collection period in 2022 was 18–28 February 2022, during the third dose of the COVID-19 vaccination program in Japan. Therefore, this study uses the 2022 dataset, which includes questions about respondents’ willingness to take the third dose of the COVID-19 vaccine as well as their demographic characteristics. Furthermore, the 2020 dataset has been used for several demographic variables such as gender, age, education, place of residence, children in the household, financial literacy, and level of risk preference. After removing missing values from control variables like household assets and household income (1369 observations), the total number of observations in this study was 2912 (68% of the total observations in the 2022 dataset).

### 2.2. Variable Definitions

The dependent variable in this study was “hesitancy towards the third dose of the COVID-19 vaccine,” which was based on a five-point scale question “I am willing to take the COVID-19 booster vaccination (3rd vaccination) when it is available free of charge.” The responses revealed the following: 1 meant “Strongly disagree,” 2 meant “Disagree,” 3 meant “Neither agree nor disagree,” 4 meant “Agree,” and 5 meant “Strongly agree.” Following Fisher et al. [39], we recoded “hesitancy towards the third dose of the COVID-19 vaccine” to a binary variable, with answers 1, 2, or 3 indicating vaccine hesitant, and answers 4 or 5 indicating not vaccine hesitant.

We included gender, age, marital status, children in the household, living condition, place of residence, education, employment status, household income, and household assets as demographic variables. Financial literacy was also incorporated into the model as a proxy for rational decision-making ability in health-related behaviors [34,40,41,42,43,44,45]. Subjective health status, anxiety about the future, myopic view of the future, and risk preference were also included in the model specifications. Table 1 provides the definitions of all the variables.

### 2.3. Methods

The relationship between the respondents’ socioeconomic factors and their apprehension towards the third dose of vaccine is determined using the following equation:

(1) $$Y_{i}=f\left(X_{i},\varepsilon_{i}\right)$$

where Y is reluctance to receive the third dose of vaccine, X is a vector of individual characteristics, and ε is the error term. The full model specification is provided in Equation (2):

(2) $$\begin{array}{c} Hesitancy\;toward\;the\;third\;dose\;of\;vaccine_{i}\;\\ \;=\beta_{0}\;+\beta_{1}male_{i}+\beta_{2}age_{i}+\beta_{3}age\;squared_{i}\\ \;+\beta_{4}spouse+\beta_{5}children_{i}+\beta_{6}living\;alone_{i}\\ \;+\beta_{7}living\;in\;central\;area_{i}+\beta_{8}university\;degree_{i}\\ \;+\beta_{9}employed_{i}+\beta_{10}\log ofhousehold\;income_{i}\\ \;+\beta_{11}\log ofhousehold\;assets_{i}+\beta_{12}financial\;literacy_{i}\\ \;+\beta_{13}subjective\;health\;status_{i}\\ \;+\beta_{14}anxiety\;about\;the\;future_{i}\\ \;+\;\beta_{15}myopic\;view\;of\;the\;future_{i}\\ \;+\beta_{16}level\;of\;risk\;preference_{i} \end{array}$$

Since the dependent variable was a binary variable, probit regression was used to estimate Equation (2).

We conducted correlation and multicollinearity tests (results available upon request) as there was a possibility of multicollinearity between the explanatory variables in the models (for example, individuals with a high level of education could have high financial literacy). The correlation matrix revealed a weak relationship between the explanatory variables (lower than 0.70). Furthermore, the variance inflation factor tests of the explanatory variables were all less than 10, indicating that multicollinearity is not significant.

## 3. Results

### 3.1. Descriptive Statistics

Descriptive statistics of the main variables are presented in Table 2. There were 2912 observations in total, with 34% of respondents expressing apprehension about receiving the third dose of vaccine. In terms of demographics, 57% of the sample was male, with an average age of 50.40 years. In terms of household status, 66% of the sample had a spouse, 54% had children, 20% lived alone, and 62% lived in the central area (around Tokyo and Osaka metropolises). A university degree was obtained by approximately 56% of the sample, and 63% were currently employed. The average annual household income was JPY 6.38 million, and the average household asset was JPY 22.50 million. The average financial literacy of respondents was 0.60. On average, respondents rated their subjective health, anxiety about the future, and myopic view of the future at 3.27, 3.81, and 2.72 out of 5, respectively. Overall, the risk preference score of respondents was 45%, indicating that they were slightly risk averse.

Table 3 shows the level of apprehension towards the third dose of vaccine, stratified by gender and age. The test statistics show that there was no significant difference in hesitancy towards the third dose of vaccine between men and women. However, we discovered that vaccine hesitancy was higher among younger men than among older men. Furthermore, the results show that there was a significant inter-age group difference in vaccine hesitancy for men but not for women.

### 3.2. Regression Results

Table 4, Table 5, Table 6 and Table 7 present the results of probit regressions. The sample was divided based on gender and age groups. We first present the findings of female and male sub-samples in younger (aged under 65) and older (aged 65 and above) age groups, followed by a detailed analysis of sub-samples aged under 65 (aged under 35, 35–49, and 50–64 years).

According to the findings presented in Table 4, having a spouse and the respondent’s subjective health status had a significant impact on both younger and older women’s reluctance to receive the third dose of vaccine. Furthermore, we discovered that employment status, household assets, anxiety about the future, and a myopic view of the future all had an impact on younger women’s reluctance to receive the third dose of vaccine.

We divided the sample of younger women into three groups based on age for further analysis. Table 5 shows that anxiety about the future had a negative and significant impact on younger women’s reluctance to receive the third dose of vaccine across all age groups. This indicated that younger women who were anxious about their future were less vaccine hesitant than their counterparts. Moreover, we discovered that having a spouse, children, and subjective health status had a greater impact on vaccine hesitancy in the youngest age group; subjective health status and myopic view of the future had a greater impact on vaccine hesitancy in the 35–49 age group; and household assets had a greater impact on vaccine hesitancy in the 50–64 age group.

Table 6 shows that having a spouse, subjective health status, and future anxiety all had a significant impact on hesitancy towards the third dose of vaccine in both younger and older men. Furthermore, we discovered that age and household assets had a greater influence on vaccine hesitancy in younger men, while household income had a greater influence on vaccine hesitancy in older men.

We divided the sample of younger women into three groups based on age for further analysis. Table 7 shows that having a spouse and subjective health status had a negative and significant impact on the reluctance to receive the third dose of vaccine across all age groups. These results indicated that younger men who had a spouse and considered themselves healthy were less vaccine hesitant than their counterparts. Furthermore, we discovered that having children, household income, and a myopic view of the future had a greater influence on vaccine hesitancy in the youngest age group; university degree, household assets, and future anxiety had a greater impact on vaccine hesitancy in the 35–49 age group; and household assets and future anxiety had a greater impact on vaccine hesitancy in the 50–64 age group.

## 4. Discussion

The mass vaccination program for the third dose of the COVID-19 vaccine has been underway in Japan, with the younger population being prioritized as the majority of the older people have already been vaccinated. The success of the third dose of the vaccine program is critically dependent on the attitude of the younger generation, as this group previously demonstrated the most apprehension towards vaccination for the first two doses in Japan [7,34,35,36,37] and many other countries of the world [46,47,48]. The issue of vaccine hesitancy for the third dose has been more challenging to address owing to the emergence of new elements of hesitancy, such as uncertainty regarding the necessity of an additional dose after being “fully vaccinated” and vaccine experience for the first two doses [4,22]. We investigated the magnitude of vaccine hesitancy among various age and gender strata of younger populations, as well as the relationship between vaccine hesitancy and demographic and socio-economic factors.

### 4.1. Vaccine Hesitancy among Younger Generation

Our findings demonstrate that reluctance to receive the third dose of the COVID-19 vaccine is widespread among the Japanese population. The youngest men were found to be the most apprehensive about receiving the third dose of vaccine. The inter-age group difference in vaccine hesitancy, on the other hand, is statistically significant only for men and not for women. The higher vaccine hesitancy among the youngest men is consistent with the global findings including Japan [7,34,35,36,37,46,47,48] as well as the fact that Japan is traditionally a vaccine hesitant country [49,50]. Furthermore, because they are fully vaccinated and may not be immunocompromised, young men may be perplexed about the need for the third dose of vaccine. The younger generation, in general, has been found to have a lower risk perception than others. However, in contrast to previous findings, younger women were not found to have significantly higher vaccine hesitancy than their older counterparts.

### 4.2. Socioeconomic and Behavioral Causes of Vaccine Hesitancy among Younger Generation

The descriptive statistics and mean variance tests demonstrate the prevalence of hesitancy towards the third dose of vaccine among the Japanese population, as well as the difference between age-based subsamples. Therefore, we conducted a probit regression analysis on various subsamples of younger generation to better understand the socioeconomic and behavioral causes of vaccine hesitancy for each group. As mentioned earlier, we did not investigate vaccine hesitancy among the older generation because the majority of them are already vaccinated.

The two most significant causes of vaccine hesitancy among men and women of various ages are anxiety about the future and subjective health status. However, the strength of the results differs across the age groups. Younger men of all ages, who were confident about their health condition were less vaccine hesitant than others. Similarly, subjective health status is a significant predictor of reluctance to receive the third dose of vaccine for younger women aged 49 and under, implying that younger women with higher confidence in their health status are less vaccine hesitant. Higher vaccine hesitancy among younger men and women with lower confidence in their health status is consistent with the findings of Soares et al. [51], who also discovered higher vaccine hesitancy among people with lower perceived health status. We posit that people who are confident about their health status will want to stay healthy by receiving the third dose of vaccine and will emphasize less on the complexities of vaccination.

Anxiety about the future has been found to be a significant factor associated with hesitancy towards the third dose of vaccine among younger women of all ages and younger men aged 35 to 64. Therefore, younger men and women who are anxious about their future would be less vaccine hesitant as they may want to live a free life by being fully vaccinated. The pandemic has had such a significant impact on the social and economic lives of younger people that they are more concerned about their health, social and economic conditions, and want to restore normalcy using all available means. Soares et al. [51] also found that anxiety, agitation, and sadness were significantly associated with vaccine hesitancy.

Furthermore, few socio-economic issues are found to be associated with the aversion to the third dose of vaccine. For example, younger men of all ages and the youngest women group who are currently married are less likely to be vaccine hesitant. Married people may be more willing to receive vaccines if they are responsible for their family. Since full vaccination is now a requirement for receiving and providing necessary services, people with family responsibilities are more likely be vaccinated to avoid unnecessary complications in their daily and social lives. Moreover, married people would like to return to normal life after being vaccinated as the pandemic has substantially compromised their social lives. Apart from the marital status, having children is negatively associated with vaccine hesitancy for the third dose among younger men and women aged 35 and under. According to our findings, younger people are also more likely to get vaccinated for the sake of their children’s safety and well-being. The value of household assets is found to be negatively associated with vaccine hesitancy among younger men aged 35 to 64 and younger women aged 50 to 64, implying that younger people with low household assets have a higher vaccine hesitancy. This finding is consistent with that of Bertoncello et al. [52], who claimed that higher vaccine hesitancy exists among people having lower socioeconomic status.

Finally, we discovered that a few other factors were inconsistently and weakly associated with the vaccine hesitancy. For example, household income, myopic view of the future, and university degree all have some bearing on vaccination decisions among younger men and women.

## 5. Conclusions

We investigated vaccine aversion among the younger generation and its causes during the ongoing mass vaccination program for the third dose of COVID-19 vaccine in Japan. According to our findings, a sizable proportion of younger men and women are still reluctant to receive the third dose of the vaccine. Furthermore, vaccine hesitancy differs significantly among men of different ages, but this evidence is not significant for women. Our probit regression results show that subjective health status and anxiety about the future are the two most significant factors associated with youths’ hesitance to receive the third dose of vaccine. However, there are few socio-economic and behavioral factors associated with vaccine hesitancy in men and women of specific age groups. The findings of this study suggest that effective communication strategies tailored to specific age groups are required to reduce apprehension about receiving the third dose of vaccine.

Our study has some limitations. First, we had to exclude several observations due to missing values for important socioeconomic variables such as household assets and income, and financial literacy. Second, because our study is based on an internet survey, respondents may be of a higher socioeconomic status, as internet access rates very by socioeconomic groups [53]. Moreover, the age and gender distribution of this study’s respondents differs slightly from national statistics [54]. Third, the small sample size in some gender- and age-based subsamples could influence the study results. Finally, the use of self-reported questions in the survey could also affect the study results. Nevertheless, our study provides comprehensive evidence of aversion to the third dose of vaccine and its underlying causes among younger women and men in Japan.

## Figures and Tables

**Table 1:** Variable definitions.

Variable	Definition
Dependent variable	
Hesitancy towards the third dose of the COVID-19 vaccine	Binary variable: 1 = Strongly disagree, disagree, neither agree nor disagree for the statement “I am willing to take the COVID-19 booster vaccination (3rd vaccination) when it is available free of charge.” and 0 = Otherwise
Explanatory variables	
Male *	Binary variable: 1 = Male and 0 = Female
Age *	Continuous variable: Respondent’s age
Age squared *	Continuous variable: Respondent’s age squared
Spouse	Binary variable: 1 = Currently married and 0 = Otherwise
Children *	Binary variable: 1 = Have child/children and 0 = Otherwise
Living alone	Binary variable: 1 = Living alone and 0 = Otherwise
Living in central area *	Binary variable: 1 = Live in Kanto (around Tokyo metropolis) and Kinki (around Osaka metropolis) areas and 0 = Otherwise
University degree *	Binary variable: 1 = Obtained university degree and 0 = Otherwise
Employed	Binary variable: 1 = Respondent is employed and 0 = otherwise
Household income	Continuous variable: Annual earned income (in JPY) before taxes and with bonuses of entire household in 2021
Log of household income	Log (household income)
Household assets	Continuous variable: Balance of financial assets (savings, stocks, bonds, insurance, etc.) of entire household (in JPY)
Log of household assets	Log (household assets)
Financial literacy *	Continuous variable: Average score of correct answers from three financial literacy questions
Subjective health status	Ordinal variable: Based on the statement “I am now healthy and was generally healthy in last 1 year.” 1 = It does not hold true at all for you; 2 = It is not so true for you; 3 = Neither true nor not true; 4 = It is rather true for you; 5 = It is particularly true for you
Anxiety about the future	Ordinal variable: It measures respondents’ anxiety over health and livelihood in the future based on the statement “I have anxieties about my ‘life after I am 65 years old’ (For those who are already aged 65 or above, ‘life in future’)” 1 = It does not hold true at all for you; 2 = It is not so true for you; 3 = Neither true nor not true; 4 = It is rather true for you; 5 = It is particularly true for you
Myopic view of the future	Ordinal variable: It measures respondents’ perception on the present compared to the future based on the statement “Since future is uncertain, it is a waste to think about it.” 1 = Completely disagree; 2 = Disagree; 3 = Neither agree nor disagree; 4 = Agree; 5 = Completely agree
Level of risk preference *	Continuous variable: Percentage score from the question “Usually when you go out, how high does the probability of rain have to be before you take an umbrella?”

Note: The symbol * indicates variables sourced from the 2020 dataset; Source: Authors.

**Table 2:** Descriptive statistics.

Variable	Mean	SD *	Min	Max
Hesitancy towards the third dose of vaccine	0.34	0.47	0	1
Male	0.57	0.49	0	1
Age	50.40	14.35	22	90
Age squared	2745.47	1489.00	484	8100
Spouse	0.66	0.47	0	1
Children	0.54	0.50	0	1
Living alone	0.20	0.40	0	1
Living in central area	0.62	0.49	0	1
University degree	0.56	0.50	0	1
Employed	0.63	0.48	0	1
Household income	6,381,868	4,204,489	500,000	21,000,000
Log of household income	15.42	0.79	13.12	16.86
Household assets	22,500,000	30,800,000	1,250,000	125,000,000
Log of household assets	16.01	1.45	14.04	18.64
Financial literacy	0.60	0.38	0	1
Subjective health status	3.27	1.13	1	5
Anxiety about the future	3.81	1.15	1	5
Myopic view of the future	2.72	1.00	1	5
Level of risk preference	0.45	0.23	0	1
Observations	2912			

Note: * SD denotes standard deviation.

**Table 3:** Hesitancy towards the third dose of COVID-19 vaccine, stratified by gender and age.

Hesitancy towards the Third Dose of Vaccine	Female				Male				Total
	Age < 35	Age 35–49	Age 50–64	Age ≥ 65	Age < 35	Age 35–49	Age 50–64	Age ≥ 65	
Non-hesitant	239	308	199	66	80	338	405	297	1932
	67.90%	65.81%	64.82%	58.93%	60.15%	71.16%	64.80%	67.50%	66.35%
Hesitant	113	160	108	46	53	137	220	143	980
	32.10%	34.19%	35.18%	41.07%	39.85%	28.84%	35.20%	32.50%	33.65%
Total	352	468	307	112	133	475	625	440	2912
	100%	100%	100%	100%	100%	100%	100%	100%	100%
Mean difference (by age group)	F = 1.04				F = 2.65 **				
Mean difference (overall)	F = 1.67								

Note: ** *p* < 0.05.

**Table 4:** Results of probit regression analysis regarding younger and older females’ reluctance to receive the third dose of COVID-19 vaccine.

Variable	Female: Age < 65	Female: Age ≥ 65
Age	0.00930	−0.166
	(0.0305)	(0.642)
Age squared	−0.0000595	0.00110
	(0.000355)	(0.00434)
Spouse	−0.257 **	−0.660 *
	(0.118)	(0.393)
Children	−0.117	−0.0239
	(0.0834)	(0.361)
Living alone	0.0600	−0.204
	(0.136)	(0.437)
Living in central area	0.0623	−0.0327
	(0.0816)	(0.294)
University degree	−0.0749	−0.251
	(0.0858)	(0.320)
Employed	0.191 **	0.379
	(0.0936)	(0.305)
Log of household income	−0.0473	0.148
	(0.0626)	(0.235)
Log of household assets	−0.0950 ***	−0.0756
	(0.0326)	(0.0966)
Financial literacy	−0.156	−0.158
	(0.111)	(0.380)
Subjective health status	−0.158 ***	−0.311 **
	(0.0364)	(0.123)
Anxiety about the future	−0.212 ***	−0.0535
	(0.0367)	(0.130)
Myopic view of the future	0.0712 *	0.00715
	(0.0418)	(0.119)
Level of risk preference	−0.217	−0.226
	(0.190)	(0.684)
Constant	2.927 ***	6.637
	(1.100)	(24.42)
Observations	1127	112
Log pseudolikelihood	−677.7	−68.58
Wald chi^2^	80.53	15.64
*p*−value	0.000	0.407
Pseudo R2	0.0600	0.0957

Note: Robust standard errors are in parentheses; *** *p* < 0.01, ** *p* < 0.05, * *p* < 0.1.

**Table 5:** Results of probit regression analysis regarding younger females’ reluctance to receive the third dose of COVID-19 vaccine.

Variable	Female: Age < 35	Female: Age 35–49	Female: Age 50–64
Age	−0.279	0.200	0.0499
	(0.376)	(0.317)	(0.538)
Age squared	0.00499	−0.00233	−0.000375
	(0.00656)	(0.00374)	(0.00479)
Spouse	−0.528 **	−0.151	−0.129
	(0.231)	(0.179)	(0.221)
Children	−0.327 *	−0.0257	−0.118
	(0.173)	(0.125)	(0.159)
Living alone	−0.162	0.201	0.107
	(0.257)	(0.209)	(0.268)
Living in central area	0.00669	0.180	−0.100
	(0.150)	(0.126)	(0.165)
University degree	−0.0885	−0.112	0.0375
	(0.162)	(0.132)	(0.175)
Employed	0.250	0.102	0.273
	(0.181)	(0.148)	(0.171)
Log of household income	−0.0573	−0.0860	0.0334
	(0.118)	(0.0965)	(0.120)
Log of household assets	−0.0974	−0.0685	−0.146 **
	(0.0612)	(0.0501)	(0.0626)
Financial literacy	−0.124	−0.152	−0.219
	(0.210)	(0.170)	(0.212)
Subjective health status	−0.118 *	−0.221 ***	−0.103
	(0.0688)	(0.0577)	(0.0678)
Anxiety about the future	−0.252 ***	−0.204 ***	−0.188 **
	(0.0660)	(0.0566)	(0.0737)
Myopic view of the future	0.0183	0.117*	0.0721
	(0.0736)	(0.0681)	(0.0793)
Level of risk preference	−0.337	−0.219	−0.120
	(0.337)	(0.309)	(0.366)
Constant	7.673	−0.957	0.856
	(5.749)	(6.833)	(15.25)
Observations	352	468	307
Log pseudolikelihood	−203.9	−279.1	−187.5
Wald chi^2^	33.81	43.89	20.51
*p*−value	0.00363	0.000114	0.153
Pseudo R2	0.0773	0.0715	0.0581

Note: Robust standard errors are in parentheses; *** *p* < 0.01, ** *p* < 0.05, * *p* < 0.1.

**Table 6:** Results of probit regression analysis regarding younger and older males’ reluctance to receive the third dose of COVID-19 vaccine.

Variable	Male: Age < 65	Male: Age ≥ 65
Age	−0.0655 **	0.430
	(0.0312)	(0.298)
Age squared	0.000728 **	−0.00286
	(0.000336)	(0.00200)
Spouse	−0.497 ***	−0.336 *
	(0.115)	(0.173)
Children	−0.0904	0.131
	(0.0803)	(0.178)
Living alone	0.0412	−0.234
	(0.129)	(0.213)
Living in central area	0.000614	−0.00472
	(0.0801)	(0.141)
University degree	0.136	0.0239
	(0.0848)	(0.138)
Employed	−0.0404	0.117
	(0.0912)	(0.148)
Log of household income	0.0596	−0.181 *
	(0.0663)	(0.106)
Log of household assets	−0.141 ***	−0.0857
	(0.0318)	(0.0528)
Financial literacy	0.0154	−0.160
	(0.109)	(0.216)
Subjective health status	−0.163 ***	−0.221 ***
	(0.0345)	(0.0559)
Anxiety about the future	−0.154 ***	−0.104 *
	(0.0362)	(0.0582)
Myopic view of the future	0.0452	0.0571
	(0.0392)	(0.0645)
Level of risk preference	0.156	0.188
	(0.160)	(0.313)
Constant	3.475 ***	−11.34
	(1.157)	(11.10)
Observations	1233	440
Log pseudolikelihood	−728.8	−256.2
Wald chi^2^	107.1	37.67
*p*−value	0	0.00101
Pseudo R2	0.0706	0.0766

Note: Robust standard errors are in parentheses; *** *p* < 0.01, ** *p* < 0.05, * *p* < 0.1.

**Table 7:** Results of probit regression analysis regarding younger males’ reluctance to receive the third dose of COVID-19 vaccine.

Variable	Male: Age < 35	Male: Age 35–49	Male: Age 50–64
Age	0.459	−0.434	−0.488
	(0.597)	(0.339)	(0.373)
Age squared	−0.00849	0.00518	0.00438
	(0.0104)	(0.00396)	(0.00329)
Spouse	−0.722 *	−0.413 **	−0.526 ***
	(0.384)	(0.208)	(0.153)
Children	−0.632 *	−0.182	−0.00252
	(0.365)	(0.128)	(0.113)
Living alone	0.380	0.191	−0.0669
	(0.449)	(0.226)	(0.170)
Living in central area	0.0426	−0.144	0.0581
	(0.245)	(0.131)	(0.114)
University degree	0.171	0.265 *	0.0677
	(0.292)	(0.143)	(0.118)
Employed	−0.350	−0.0381	−0.00973
	(0.281)	(0.149)	(0.130)
Log of household income	0.462 **	0.0725	−0.000762
	(0.216)	(0.106)	(0.0969)
Log of household assets	−0.0799	−0.117 **	−0.186 ***
	(0.0950)	(0.0512)	(0.0465)
Financial literacy	0.300	0.104	−0.0918
	(0.338)	(0.194)	(0.149)
Subjective health status	−0.237 **	−0.125 **	−0.173 ***
	(0.117)	(0.0542)	(0.0498)
Anxiety about the future	−0.117	−0.184 ***	−0.153 ***
	(0.120)	(0.0569)	(0.0527)
Myopic view of the future	0.234 *	0.0286	0.0224
	(0.132)	(0.0642)	(0.0558)
Level of risk preference	−0.119	0.116	0.228
	(0.532)	(0.265)	(0.228)
Constant	−11.21	10.35	17.42
	(8.806)	(7.366)	(10.60)
Observations	133	475	625
Log pseudolikelihood	−79.01	−265.1	−372.8
Wald chi^2^	20.65	44.27	60.86
*p*−value	0.149	0.000	0.000
Pseudo R2	0.117	0.0708	0.0805

Note: Robust standard errors are in parentheses; *** *p* < 0.01, ** *p* < 0.05, * *p* < 0.1.

## Data Availability

Data is available upon request.

## References

[1] Latkin C.A., Dayton L., Yi G., Konstantopoulos A., Boodram B. (2021). Trust in a COVID-19 Vaccine in the US: A Social-Ecological Perspective. Soc. Sci. Med..

[2] Joshi A., Kaur M., Kaur R., Grover A., Nash D., El-Mohandes A. (2021). Predictors of COVID-19 Vaccine Acceptance, Intention, and Hesitancy: A Scoping Review. Front. Public Health.

[3] Wang Y., Liu Y. (2021). Multilevel determinants of COVID-19 vaccination hesitancy in the United States: A rapid systematic review. Prev. Med. Rep..

[4] Babicki M., Mastalerz-Migas A. (2022). Attitudes of Poles Towardss the COVID-19 Vaccine Booster Dose: An Online Survey in Poland. Vaccines.

[5] Cerda A.A., García L.Y. (2021). Hesitation and Refusal Factors in Individuals’ Decision-Making Processes Regarding a Coronavirus Disease 2019 Vaccination. Front. Public Health.

[6] Biswas N., Mustapha T., Khubchandani J., Price J.H. (2021). The Nature and Extent of COVID-19 Vaccination Hesitancy in Healthcare Workers. J. Commun. Health.

[7] Kadoya Y., Watanapongvanich S., Yuktadatta P., Putthinun P., Lartey S.T., Khan M.S.R. (2021). Willing or Hesitant? A Socioeconomic Study on the Potential Acceptance of COVID-19 Vaccine in Japan. Int. J. Environ. Res. Public Health.

[8] Bell S., Clarke R., Mounier-Jack S., Walker J.L., Paterson P. (2020). Parents’ and Guardians’ Views on the Acceptability of a Future COVID-19 Vaccine: A Multi-Methods Study in England. Vaccine.

[9] Walkowiak M.P., Walkowiak D. (2021). Predictors of COVID-19 Vaccination Campaign Success: Lessons Learnt from the Pandemic so Far. A Case Study from Poland. Vaccines.

[10] Ferdinands J.M., Rao S., Dixon B.E., Mitchell P.K., DeSilva M.B., Irving S.A., Lewis N., Natarajan K., Stenehjem E., Grannis S.J. (2022). Waning 2-Dose and 3-Dose Effectiveness of mRNA Vaccines Against COVID-19–Associated Emergency Department and Urgent Care Encounters and Hospitalizations Among Adults During Periods of Delta and Omicron Variant Predominance—VISION Network, 10 States, August 2021–January 2022. MMWR Morb. Mortal. Wkly Rep..

[11] FDA (2021). FDA Authorizes Booster Dose of Pfizer-BioNTech COVID-19 Vaccine for Certain Populations. https://www.fda.gov/news-events/press-announcements/fda-authorizes-booster-dose-pfizer-biontech-covid-19-vaccine-certain-populations.

[12] The Japan Times (2021). Japan Prepares Booster Shots Amid Worries over Waning Vaccine Efficacy. https://www.japantimes.co.jp/news/2021/09/23/national/booster-shot-japan-explainer/.

[13] Shrotri M., Navaratnam A.M.D., Nguyen V., Byrne T., Geismar C., Fragaszy E., Beale S., Fong W.L.E., Patel P., Kovar J. (2021). Spike-Antibody Waning After Second Dose of BNT162b2 or ChAdOx1. Lancet.

[14] Yamayoshi S., Yasuhara A., Ito M., Akasaka O., Nakamura M., Nakachi I., Koga M., Mitamura K., Yagi K., Maeda K. (2021). Antibody Titers Against SARS-CoV-2 Decline, but Do Not Disappear for Several Months. EClinicalmedicine.

[15] Liu Y., Rocklöv J. (2021). The Reproductive Number of the Delta Variant of SARS-CoV-2. Is Far Higher Compared to the Ancestral SARS-CoV-2 Virus. J. Travel Med..

[16] Tang P., Hasan M.R., Chemaitelly H., Yassine H.M., Benslimane F.M., Khatib H.A.A., AlMukdad S., Coyle P., Ayoub H.H., Kanaani Z.A. (2021). BNT162b2 and mRNA-1273 COVID-19 Vaccine Effectiveness Against the Delta (B.1.617.2) Variant in Qatar. Nat. Med..

[17] Chia P.Y., Ong S.W.X., Chiew C.J., Ang L.W., Chavatte J.-M., Mak T.-M., Cui L., Kalimuddin S., Chia W.N., Tan C.W. (2022). Virological and serological kinetics of SARS-CoV-2 Delta variant vaccine-breakthrough infections: A multi-center cohort study. Clin. Microbiol. Infect..

[18] Hall V.G., Ferreira V.H., Ku T., Ierullo M., Majchrzak-Kita B., Chaparro C., Selzner N., Schiff J., McDonald M., Tomlinson G. (2021). Randomized Trial of a Third Dose of mRNA-1273 Vaccine in Transplant Recipients. N. Engl. J. Med..

[19] Kamar N., Abravanel F., Marion O., Couat C., Izopet J., Del Bello A. (2021). Three Doses of an mRNA COVID-19 Vaccine in Solid-Organ Transplant Recipients. N. Engl. J. Med..

[20] Batra K., Sharma M., Dai C.-L., Khubchandani J. (2022). COVID-19 Booster Vaccination Hesitancy in the United States: A Multi-Theory-Model (MTM)-Based National Assessment. Vaccines.

[21] Folcarelli L., Miraglia Del Giudice G., Corea F., Angelillo I.F. (2022). Intention to Receive the COVID-19 Vaccine Booster Dose in a University Community in Italy. Vaccines.

[22] Rzymski P., Poniedziałek B., Fal A. (2021). Willingness to Receive the Booster COVID-19 Vaccine Dose in Poland. Vaccines.

[23] Yadete T., Batra K., Netski D.M., Antonio S., Patros M.J., Bester J.C. (2021). Assessing Acceptability of COVID-19 Vaccine Booster Dose among Adult Americans: A Cross-Sectional Study. Vaccines.

[24] Klugar M., Riad A., Mohanan L., Pokorná A. (2021). COVID-19 Vaccine Booster Hesitancy (VBH) of Healthcare Workers in Czechia: National Cross-Sectional Study. Vaccines.

[25] Pal S., Shekhar R., Kottewar S., Upadhyay S., Singh M., Pathak D., Kapuria D., Barrett E., Sheikh A.B. (2021). COVID-19 Vaccine Hesitancy and Attitude Towards Booster Doses Among US Healthcare Workers. Vaccines.

[26] Jairoun A.A., Al-Hemyari S.S., El-Dahiyat F., Jairoun M., Shahwan M., Al Ani M., Habeb M., Babar Z.U. (2022). Assessing Public Knowledge, Attitudes and Determinants of Third COVID-19 Vaccine Booster Dose Acceptance: Current Scenario and Future Perspectives. J. Pharm. Policy Pract..

[27] Sønderskov K.M., Vistisen H.T., Dinesen P.T., Østergaard S.D. (2022). A Positive Update on COVID-19 Booster Vaccine Willingness Among Danes. Dan. Med. J..

[28] Tung T.H., Lin X.Q., Chen Y., Zhang M.X., Zhu J.S. (2022). Willingness to Receive a Booster Dose of Inactivated Coronavirus Disease 2019 Vaccine in Taizhou, China. Expert Rev. Vaccines.

[29] Lounis M., Bencherit D., Rais M.A., Riad A. (2022). COVID-19 Vaccine Booster Hesitancy (VBH) and Its Drivers in Algeria: National Cross-Sectional Survey-Based Study. Vaccines.

[30] Paul E., Fancourt D. (2022). Predictors of uncertainty and unwillingness to receive the COVID-19 booster vaccine: An observational study of 22,139 fully vaccinated adults in the UK. Lancet Reg. Health Eur..

[31] Yoshida M., Kobashi Y., Kawamura T., Shimazu Y., Nishikawa Y., Omata F., Zhao T., Yamamoto C., Kaneko Y., Nakayama A. (2022). Factors Associated with COVID-19 Vaccine Booster Hesitancy: A Retrospective Cohort Study, Fukushima Vaccination Community Survey. Vaccines.

[32] Albanese M. (2022). How to Explain Third Dose Vaccine Hesitancy in Canada?. Experts Point to a Few Reasons..

[33] Dubé E., MacDonald N.E. (2022). COVID-19 Vaccine Hesitancy. Nat. Rev. Nephrol..

[34] Khan M.S.R., Watanapongvanich S., Kadoya Y. (2021). COVID-19 Vaccine Hesitancy Among the Younger Generation in Japan. Int. J. Environ. Res. Public Health.

[35] Yoda T., Katsuyama H. (2021). Willingness to Receive COVID-19 Vaccination in Japan. Vaccines.

[36] Machida M., Nakamura I., Kojima T., Saito R., Nakaya T., Hanibuchi T., Takamiya T., Odagiri Y., Fukushima N., Kikuchi H. (2021). Acceptance of a COVID-19 Vaccine in Japan During the COVID-19 Pandemic. Vaccines.

[37] Okubo R., Yoshioka T., Ohfuji S., Matsuo T., Tabuchi T. (2021). COVID-19 Vaccine Hesitancy and Its Associated Factors in Japan. Vaccines.

[38] Vaccination Recording System (2022). Inoculation Status of the New Corona Vaccine. https://info.vrs.digital.go.jp/dashboard/#overview.

[39] Fisher K.A., Bloomstone S.J., Walder J., Crawford S., Fouayzi H., Mazor K.M. (2020). Attitudes Towards a Potential SARS-CoV-2 Vaccine: A Survey of US Adults. Ann. Intern. Med..

[40] Kadoya Y., Khan M.S.R. (2018). Can Financial Literacy Reduce Anxiety about Life in Old Age?. J. Risk Res..

[41] Kadoya Y., Khan M.S.R., Hamada T., Dominguez A. (2018). Financial Literacy and Anxiety About Life in Old Age: Evidence from the USA. Rev. Econ. Household..

[42] Ono S., Yuktadatta P., Taniguchi T., Iitsuka T., Noguchi M., Tanaka S., Ito H., Nakamura K., Yasuhara N., Miyawaki C. (2021). Financial Literacy and Exercise Behavior: Evidence from Japan. Sustainability.

[43] Putthinun P., Watanapongvanich S., Khan M.S.R., Kadoya Y. (2021). Financial Literacy and Alcohol Drinking Behavior: Evidence from Japan. Sustainability.

[44] Watanapongvanich S., Khan M.S.R., Putthinun P., Ono S., Kadoya Y. (2021). Financial Literacy, Financial Education, and Smoking Behavior: Evidence from Japan. Front. Public Health.

[45] Khan M.S.R., Putthinun P., Watanapongvanich S., Yuktadatta P., Uddin M.A., Kadoya Y. (2021). Do Financial Literacy and Financial Education Influence Smoking Behavior in the United States?. Int. J. Environ. Res. Public Health.

[46] Robertson E., Reeve K.S., Niedzwiedz C.L., Moore J., Blake M., Green M., Katikireddim V.S., Benzeval J.M. (2021). Predictors of COVID-19 vaccine hesitancy in the UK household longitudinal study. Brain Behav. Immun..

[47] Shih S.F., Wagner A.L., Masters N.B., Prosser L.A., Lu Y., Zikmund-Fisher B.J. (2021). Vaccine hesitancy and rejection of a vaccine for the novel coronavirus in the United States. Front. Immunol..

[48] Razai M.S., Chaudhry U.A., Doerholt K., Bauld L., Majeed A. (2021). Covid-19 vaccination hesitancy. BMJ.

[49] De Figueiredo A., Simas C., Karafillakis E., Paterson P., Larson H.J. (2020). Mapping Global Trends in Vaccine Confidence and Investigating Barriers to Vaccine Uptake: A Large-Scale Retrospective Temporal Modelling Study. Lancet.

[50] Mahase E. (2021). Covid-19: UK Has Highest Vaccine Confidence and Japan and South Korea the Lowest, Survey Finds. BMJ.

[51] Soares P., Rocha J.V., Moniz M., Gama A., Laires P.A., Pedro A.R., Dias S., Leite A., Nunes C. (2021). Factors Associated with COVID-19 Vaccine Hesitancy. Vaccines.

[52] Bertoncello C., Ferro A., Fonzo M., Zanovello S., Napoletano G., Russo F., Baldo V., Cocchio S. (2020). Socioeconomic Determinants in Vaccine Hesitancy and Vaccine Refusal in Italy. Vaccines.

[53] Ministry of Internal Affairs and Communications (2020). Communications Usage Trend Survey in 2019 Compiled. Tokyo, Japan. http://www.soumu.go.jp/johotsusintokei/statistics/data/200529_1.pdf.

[54] Statistics Bureau (2021). Japan Statistical Yearbook, Tokyo, Japan. https://www.stat.go.jp/english/data/nenkan/70nenkan/zenbun/en70/book/book.pdf.

